# Exogenous Methyl Jasmonate Mediates Secondary Metabolic Reprogramming to Enhance Resistance in Tea Plants

**DOI:** 10.3390/plants15020311

**Published:** 2026-01-20

**Authors:** Jie Liu, Zaifa Shu, Xinyan Lan, Dayun Zhou, Huiting Yang, Huijuan Zhou, Qingyong Ji, Limin Chen, Weizhong He

**Affiliations:** 1 Lishui Key Laboratory of Tea Plant Resource Development and Utilization, Lishui Institute of Agricultural and Forestry Sciences, Lishui 323000, China; zjlsliujie@163.com (J.L.); shuzaifa@163.com (Z.S.); lssnlkyzdy@163.com (D.Z.); yht093322@163.com (H.Y.); lsszhj1020@163.com (H.Z.); healthhome@126.com (Q.J.); 2 College of Agriculture and Biotechnology, Lishui University, Lishui 323000, China; lanxya4086@163.com

**Keywords:** methyl jasmonate, *Camellia sinensis*, plant defense, bud morphology, transcriptomics

## Abstract

Tea plants are frequently threatened by insect pests, resulting in substantial yield and quality losses. Methyl jasmonate (MeJA) is a key defense signaling molecule in plants; however, its integrated effects on tea plant growth, resistance, and quality-related traits remain poorly understood. In this study, field experiments were conducted to evaluate the effects of exogenous MeJA at different concentration (0.02–20 mM) on growth traits, quality components, and resistance to the tea green leafhopper and tea orange gall mite in *Camellia sinensis* ‘Zhongcha 108’, and transcriptome analysis was further integrated to elucidate the underlying molecular mechanisms. The results showed that appropriate MeJA concentrations (0.2–2 mM) significantly optimized bud morphology, characterized by shortened internodes, thicker stems, and reduced leaf insertion angles. Importantly, these treatments did not significantly alter the measured quality-related biochemical components, such as free amino acids and soluble sugars, within the evaluated time frame. Collectively, this study provides the first field-based evidence defining an effective MeJA concentration window that balances pest resistance induction, growth modulation, and processing suitability for flat-type green tea, offering practical guidance for the rational application of MeJA in tea plantation management.

## 1. Introduction

*Camellia sinensis* is one of the most distinctive and economically important traditional agricultural industries in China. As of 2024, the national tea plantation area has reached approximately 3.4952 million hectares, with a total output value exceeding 322 billion RMB [1]. Among the six major tea categories in China, green tea holds the largest share in both production and economic value. Premium green teas, such as Xihu-Longjing, Dongting-Biluochun, and Huangshan-Maofeng, enjoy widespread domestic and international recognition. With growing consumer demand for higher quality, safety, and functionality, achieving a synergistic improvement in tea quality without compromising yield stability has become a central objective for the sustainable development of the tea industry.

In practical tea production, to meet market demand for the early commercialization of spring tea, growers frequently apply plant growth regulators such as gibberellin (GA_3_) and brassinolide (BR) to promote bud sprouting and increase early-stage yield. Previous studies have shown that GA_3_ can advance bud break and improve bud uniformity, and has been associated with enhanced theanine biosynthesis, possibly through the downregulation of *CsWRKY71*, thereby improving the fresh taste of tea leaves [2]. Brassinolide has been reported to increase bud density and the weight of one hundred buds [3]. However, inappropriate or excessive application of these growth regulators can negatively affect tea quality. For example, high concentrations of GA_3_ (75 mg·L^−1^) significantly increase the ash content of tea buds, while overuse of the compound growth regulator “Bihu” suppresses bud growth [4]. Current tea production requires effective strategies to regulate plant growth while maintaining or even improving tea quality, without relying excessively on conventional growth regulators or endogenous hormone manipulation. Accordingly, we aimed to investigate whether the exogenous application of methyl jasmonate (MeJA), a functional analog of endogenous jasmonic acid signaling, could alleviate growth–quality–defense trade-offs in tea plants when applied at appropriate concentrations under field conditions.

MeJA is a volatile methyl ester of jasmonic acid (JA) and a biologically active member of the jasmonate family. In recent years, the potential applications of jasmonates in green tea production have attracted increasing attention [5,6]. Jasmonates are involved in a wide range of physiological processes, including the regulation of plant growth and development [7], flowering [8,9], and senescence [10], and they play critical roles in plant stress resistance [11]. As key signaling molecules in the plant immune system, jasmonates are widely involved in regulating systemic defense responses to both biotic stresses, such as insect herbivory [12] and pathogen infection [13], and abiotic stresses, including drought [14], salinity [15], and low temperature [16,17].

When plants are subjected to stress, the levels of JA and jasmonoyl-isoleucine (JA-Ile) increase rapidly at damaged sites, inducing wound-triggered local and systemic defense responses [18,19,20]. In this process, JA-Ile is perceived by the jasmonate receptor CORONATINE-INSENSITIVE 1 (COI1), a core component of the SCF^COI1^ ubiquitin ligase complex [21,22]. This interaction promotes the degradation of JAZ (jasmonate-ZIM domain) proteins via the 26S proteasome, thereby releasing transcription factors such as MYC2 and its homologs to activate downstream defense-related genes. Consequently, the biosynthesis and accumulation of multiple defensive compounds, including proteinase inhibitors and polyphenol oxidases, are enhanced, ultimately improving plant resistance [6,23,24].

MeJA is a volatile derivative of jasmonates synthesized by jasmonic acid carboxyl methyltransferase [25]. Due to its high volatility, MeJA can function as an airborne or internal signal, transmitting stress cues to undamaged tissues or neighboring plants and thereby triggering systemic immune responses [11]. Previous studies have demonstrated that MeJA affects vegetative growth [26,27,28,29], reproductive development [30,31,32], and stress resistance [33]. In tea plants, exogenous MeJA application has been shown to induce resistance against the *Ectropis obliqua* by interfering with insect physiological metabolism [12]. Moreover, MeJA treatment promotes the emission of diverse volatile organic compounds, enhancing the attraction of natural enemies such as *Apanteles* spp. and thereby strengthening ecological defense capacity [12,34,35,36].

However, activation of plant defense responses is often accompanied by resource reallocation, potentially resulting in metabolic trade-offs between growth and defense or between quality formation and resistance enhancement [6,17]. During defense induction, MeJA may exert unintended effects on sensory and nutritional quality traits of tea leaves [37,38]. To date, systematic studies evaluating the concentration-dependent effects of MeJA on tea plant growth, quality formation, and field resistance particularly under realistic field conditions remain limited, and the underlying molecular mechanisms are not yet fully understood.

Based on this, the present study systematically evaluated the effects of exogenous MeJA application at different concentrations on tea plant growth morphology, key quality components, and field resistance against major pests through field spraying experiments. Combined with transcriptome sequencing, the molecular mechanisms underlying MeJA-induced systemic resistance and metabolic network reconstruction in tea plants were further elucidated. This research aims to provide a theoretical basis and practical reference for green tea plantation management and quality regulation based on plant immunity inducers.

## 2. Results

### 2.1. Effects of Exogenous Methyl Jasmonate on Tea Buds Growth

**Figure 1:**
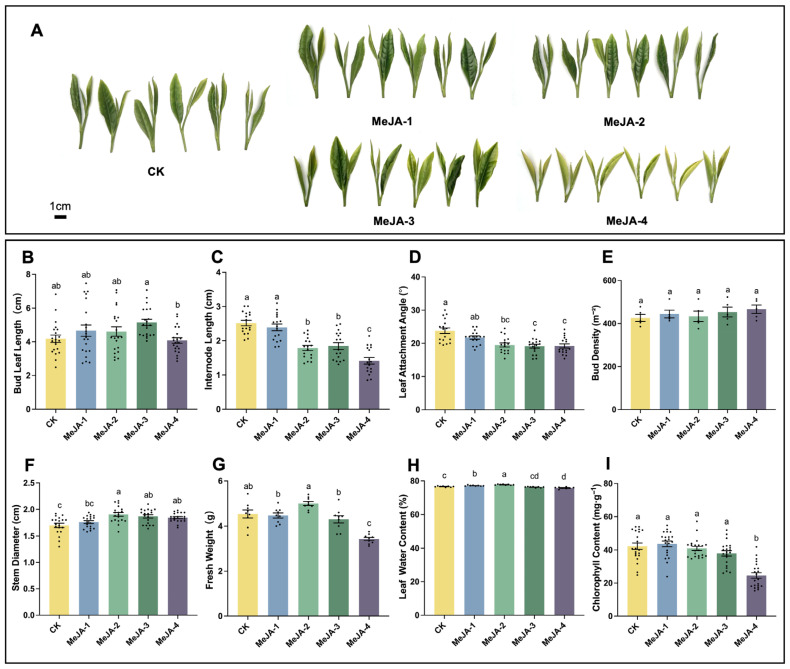
Effects of different MeJA concentrations on tea buds. (**A**) Bud growth at 14 days after the initial MeJA application. (**B**) Comparison of bud leaf length (n = 21, *F*_4,100_ = 3.022, *p* = 0.0213). (**C**) Comparison of internode length (n = 17, *F*_4,80_ = 26.05, *p* < 0.0001). (**D**) Comparison of the leaf attachment angle (n = 17, *F*_4,80_ = 10.21, *p* < 0.0001). (**E**) Comparison of bud density (n = 5, *F*_4,20_ = 0.6089, *p* = 0.6610). (**F**) Comparison of stem diameter (n = 19, *F*_4,90_ = 7.309, *p* < 0.0001). (**G**) Comparison of fresh weight (n = 9, *F*_4,40_ = 19.91, *p* < 0.0001). (**H**) Comparison of leaf water content (n = 8, *F*_4,35_ = 36.56, *p* < 0.0001). (**I**) Comparison of chlorophyll content (n = 21, *F*_4,100_ = 21.86, *p* < 0.0001). CK represents the water-treated control. MeJA-1, MeJA-2, MeJA-3, and MeJA-4 correspond to MeJA treatments at concentrations of 0.02 mM, 0.2 mM, 2 mM, and 20 mM, respectively. Bar graphs show mean ± SE, and different lowercase letters indicated significant differences among treatments (one-way ANOVA, Tukey’s HSD test, *p* < 0.05).

To investigate the effects of exogenous methyl jasmonate (MeJA) on tea plant growth, resistance and quality related traits, we examined the influence of different MeJA concentrations on tea bud development. At 14 days after the initial MeJA application, the morphology of tea buds was shown in Figure 1A. Compared with the control, none of the MeJA treatments significantly affected bud length; however, bud length under 20 mM MeJA (MeJA-4) was significantly lower than that under 2 mM MeJA (MeJA-3) (Figure 1B). Higher concentrations of MeJA (≥0.2 mM) significantly decreased internode length (Figure 1C) and the attachment angle of the two fully expanded leaves (Figure 1D), while significantly increasing stem diameter (Figure 1F). The results revealed that exogenous MeJA significantly affected tea bud morphology, whereas its effect on bud density was not significant (Figure 1E). Such morphological characteristics, including reduced internode length and smaller leaf angles, are generally considered favorable for the processing of flat-type green teas.

Regarding the fresh weight and water content of tea buds, they exhibited the same trend of change after MeJA treatment. A high MeJA concentration (20 mM) significantly reduced both the fresh weight (10 buds) and water content of tea buds, whereas an appropriate concentration of MeJA treatment (0.2 mM) significantly increased them. Regarding chlorophyll content, 20 mM MeJA significantly reduced chlorophyll levels in the two fully expanded leaves of new buds, likely reflecting inhibitory or stress-related effects under excessive hormonal stimulation. In contrast, lower MeJA concentrations (≤2 mM) did not cause significant changes compared with the control (Figure 1I).

### 2.2. Field Evaluation of Tea Plant Resistance to Empoasca onukii and Acaphylla theae After MeJA Treatment

**Figure 2:**
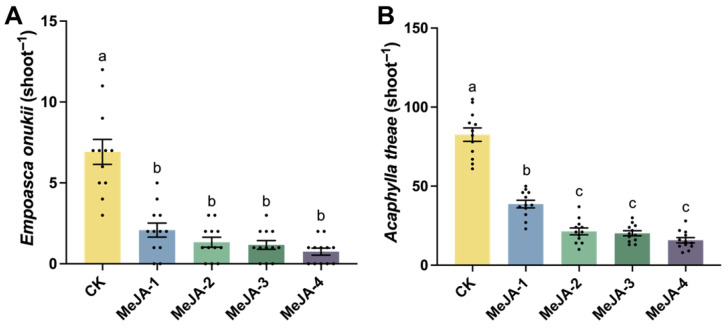
Field comparison of pest infestation levels in tea plants treated with different concentrations of MeJA. (**A**) Abundance of *E. onukii* on the second leaf (n = 12, *F*_4,55_ = 32.24, *p* < 0.0001). (**B**) Abundance of *A. theae* on the second leaf (n = 12, *F*_4,55_ = 112.6, *p* < 0.0001). Bar charts represent mean ± SE. Different lowercase letters indicated significant differences among treatments (one-way ANOVA followed by Tukey’s HSD test, *p* < 0.05).

To investigate the effects of exogenous MeJA on pest resistance in tea plants under field conditions, the population densities of *Empoasca onukii* and *Acaphylla theae* were monitored following MeJA application. The results showed that all MeJA treatments significantly reduced the infestation levels of both pests compared with the control, indicating that MeJA application effectively enhanced field resistance of tea plants to these two major herbivores (Figure 2). Notably, higher MeJA concentrations (≥0.2 mM) exhibited a stronger suppressive effect on *A*. *theae* populations than the 0.02 mM treatment. Overall, the results revealed a concentration-related trend, in which increased MeJA concentrations were generally associated with enhanced suppression of pest infestation in tea plants.

### 2.3. Effects of Exogenous MeJA on Tea Buds Nutrients, Secondary Metabolites and Resistance Enzyme Activity

**Figure 3:**
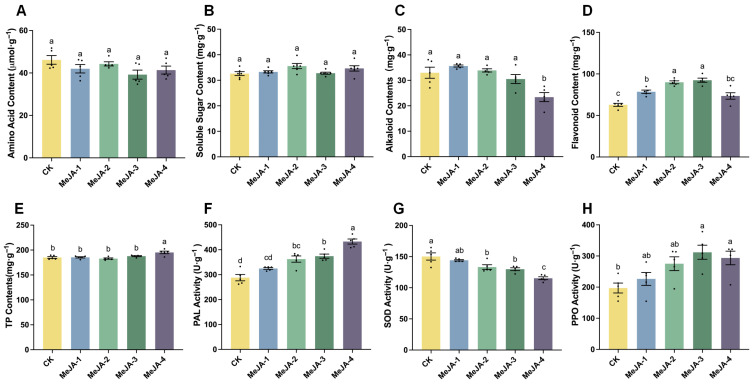
Effects of different MeJA concentrations on nutrient contents, secondary metabolites, and defense-related enzyme activities in tea leaves. (**A**) Comparison of amino acid content (n = 5, *F*_4,20_ = 2.105, *p* = 0.1181). (**B**) Comparison of soluble sugar content (n = 6, *F*_4,25_ = 2.658, *p* = 0.0564). (**C**) Comparison of alkaloid content (n = 5, *F*_4,20_ = 9.848, *p* = 0.0001). (**D**) Comparison of flavonoid content (n = 5, *F*_4,20_ = 23.18, *p* < 0.0001). (**E**) Comparison of total phenolic (TP) content (n = 5, *F*_4,20_ = 7.891, *p* = 0.0005). (**F**) Comparison of phenylalanine ammonia-lyase (PAL) activity (n = 5, *F*_4,20_ = 29.10, *p* < 0.0001). (**G**) Comparison of superoxide dismutase (SOD) activity (n = 5, *F*_4,20_ = 15.47, *p* < 0.0001). (**H**) Comparison of polyphenol oxidase (PPO) activity (n = 5, *F*_4,20_ = 5.285, *p* = 0.0045). Bar charts represent mean ± SE. Different lowercase letters indicate significant differences among treatments (one-way ANOVA, Tukey’s HSD test, *p* < 0.05).

To further clarify the potential effects of MeJA treatment on tea quality related traits, we assessed the contents of free amino acids, soluble sugars, flavonoids, and alkaloids, as well as the activities of several defense-related enzymes (including PPO and SOD), in the second fully expanded leaf from the bud apex under different MeJA concentrations. As shown in Figure 3, no significant differences were observed in free amino acid content (Figure 3A) or soluble sugar content (Figure 3B) among the treatments.

In terms of secondary metabolites associated with defense responses, MeJA-4 treatment markedly reduced alkaloid content in tea leaves (Figure 3C), whereas flavonoid levels were significantly increased under low and moderate MeJA concentrations (≤2 mM) (Figure 3D). In addition, MeJA-4 treatment substantially elevated TP content in tea leaves (Figure 3E).

Regarding defense-related enzyme activities, MeJA treatments at concentrations ≥ 0.2 mM significantly enhanced PAL activity in tea leaves (Figure 3F), while significantly reducing SOD activity (Figure 3G). Higher MeJA concentrations (≥2 mM) also significantly increased PPO activity (Figure 3H). Consistent with observations in other plant species, the MeJA-induced activation of PAL and PPO, together with enhanced flavonoid biosynthesis and accumulation, may represent key biochemical processes underlying MeJA-mediated defense induction in tea plants, which may also influence tea quality formation.

### 2.4. RNA Quality Assessment and Sequencing Data Statistics

To elucidate the molecular mechanisms underlying MeJA-induced resistance in tea plants, and based on the comprehensive effects of the various MeJA treatments on tea bud growth and development, content of internal components, field insect resistance, and levels of resistance-related substances observed in earlier stages, this study subsequently selected MeJA-2 and MeJA-3 for further transcriptome sequencing research. Transcriptome sequencing was performed on samples from the control (CK), MeJA-2, and MeJA-3 treatments, with three biological replicates per treatment (9 samples in total). After filtering and quality control, the number of clean reads obtained per sample ranged from 65,082,118 to 82,571,356 in the CK group, 63,990,324 to 67,252,488 in the MeJA-2 group, and 63,788,598 to 76,421,390 in the MeJA-3 group. In total, approximately 92.91 Gb of clean bases were generated through paired-end sequencing, with no sample yielding less than 9.7 Gb of sequencing data. The percentage of bases with Q30 scores was ≥96%, and the GC content ranged from 43.65% to 45.55%. The overall mapping rate against the reference tea genome ranged from 90.84% to 91.81% (Table 1). These quality metrics indicate that the sequencing data were of high quality and suitable for downstream analyses, including differential gene expression analysis.

### 2.5. Transcriptome Sequencing Analysis

**Figure 4:**
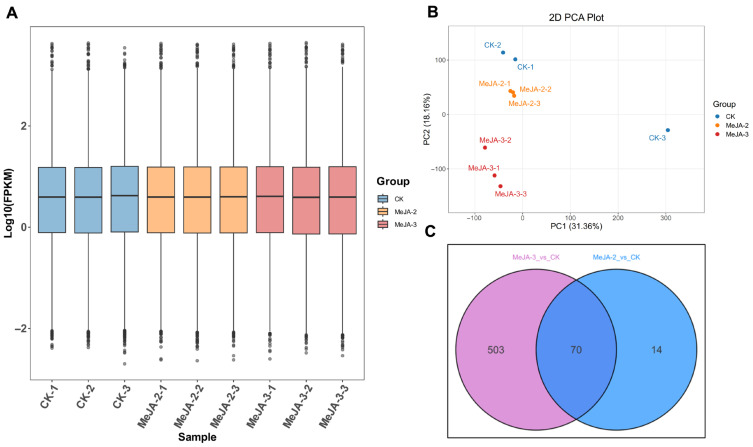
Transcriptome analysis of tea leaves after MeJA treatment. (**A**) Violin plots of gene expression levels in different treatment groups. (**B**) Principal component analysis (PCA) of transcriptomic profiles from control (CK) and MeJA-treated samples. (**C**) Numbers of differentially expressed genes (DEGs) in each MeJA treatment group.

To investigate the effects of MeJA treatment on gene expression in tea plants, we first examined the distribution of FPKM (fragments per kilobase of transcript per million mapped reads) values across samples. All biological replicates showed highly consistent overall expression patterns (Figure 4A), indicating good experimental reproducibility. Principal component analysis (PCA) revealed a clear separation among the treatment groups along principal component 2 (PC2, explaining 18.16% of the variance) (Figure 4B), suggesting that MeJA application induced systematic transcriptomic alterations.

Differential expression analysis identified 84 differentially expressed genes (DEGs) in the MeJA-2 treatment compared with the control, including 24 upregulated genes (Table S1). In the MeJA-3 treatment, 573 DEGs were detected, of which 170 were upregulated (Table S2). A total of 70 DEGs were shared between the two MeJA treatments (Figure 4C). To further elucidate the biological processes and pathways regulated by MeJA, Gene Ontology (GO) annotation and Kyoto Encyclopedia of Genes and Genomes (KEGG) pathway enrichment analyses were performed on these DEGs.

### 2.6. GO Enrichment Analysis

**Figure 5:**
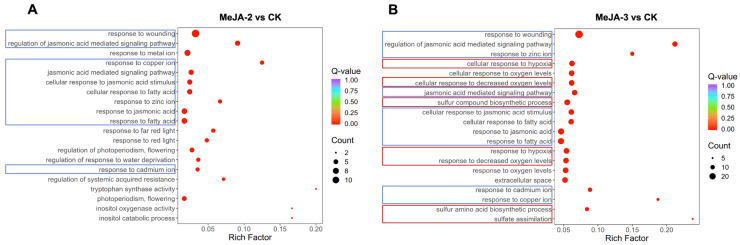
GO enrichment analysis of differentially expressed genes in tea plants under different concentrations of MeJA treatment. (**A**) MeJA-2 vs. CK. (**B**) MeJA-3 vs. CK. Blue boxes denote pathways commonly enriched in both MeJA-2 and MeJA-3, while red boxes denote pathways specifically enriched in MeJA-3 relative to MeJA-2. Boxed pathways represent selected pathways discussed in the text rather than the complete set of enriched terms.

Gene Ontology (GO) enrichment analysis was performed on differentially expressed genes (DEGs) identified from comparisons between MeJA treated samples (MeJA-2 and MeJA-3) and the control (CK). The results revealed significant enrichment of multiple Biological Process (BP) categories related to jasmonic acid-associated responses in both MeJA treatments. These included response to jasmonic acid, cellular response to jasmonic acid stimulus, jasmonic acid mediated signaling pathway, and response to fatty acid (Figure 5, blue). In addition, several stress-associated GO terms, such as response to wounding and responses to copper ion, cadmium ion, and zinc ion, were also significantly enriched. These categories are commonly associated with general stress signaling, detoxification processes, and redox regulation, suggesting activation of transcriptional programs consistent with known MeJA responsive and defense related pathways.

Compared with MeJA-2, the MeJA-3 treatment uniquely enriched GO terms related to hypoxia associated responses (e.g., cellular response to hypoxia and response to hypoxia) and sulfur-related metabolic processes, including sulfur compound biosynthetic process and sulfate assimilation (Figure 5, red). The enrichment of these categories suggests that higher MeJA concentration (2 mM) elicited a broader range of transcriptional responses, potentially reflecting enhanced metabolic and redox adjustments rather than direct hypoxic stress. Together, these results indicate that increasing MeJA concentration leads to more extensive transcriptional reprogramming in tea plants.

### 2.7. KEGG Pathway Analysis

**Figure 6:**
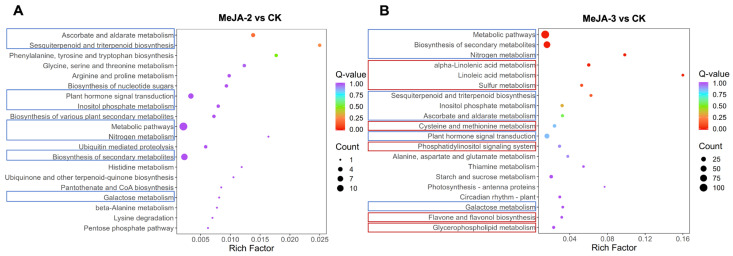
KEGG pathway enrichment analysis of differentially expressed genes in tea plants under different concentrations of MeJA treatment. (**A**) MeJA-2 vs. CK. (**B**) MeJA-3 vs. CK. Blue boxes indicate pathways significantly enriched in both MeJA-2 and MeJA-3 treatments, whereas red boxes denote pathways uniquely enriched in the MeJA-3 treatment. Boxed pathways represent selected pathways discussed in the text rather than the complete set of enriched terms.

Kyoto Encyclopedia of Genes and Genomes (KEGG) pathway enrichment analysis was performed to further characterize the biological pathways regulated by MeJA treatment. Among the top 20 enriched pathways, eight pathways were significantly enriched in both MeJA-2 and MeJA-3 treatments (Figure 6, blue). These commonly enriched pathways included metabolic pathways, biosynthesis of secondary metabolites, nitrogen metabolism, sesquiterpenoid and triterpenoid biosynthesis, inositol phosphate metabolism, ascorbate and aldarate metabolism, plant hormone signal transduction, and galactose metabolism, indicating that MeJA treatment broadly affected primary metabolism, secondary metabolite biosynthesis, and hormone-related signaling.

In contrast, the MeJA-3 treatment uniquely showed significant enrichment of several pathways closely associated with biotic and abiotic stress responses (Figure 6, red), including α-linolenic acid metabolism, linoleic acid metabolism, sulfur metabolism, cysteine and methionine metabolism, and flavone and flavonol biosynthesis. Notably, α-linolenic acid metabolism is a key pathway involved in jasmonic acid biosynthesis, and its enrichment is consistent with enhanced jasmonate-associated signaling under higher MeJA concentration. Overall, these results suggest that a higher concentration of MeJA (2 mM) is associated with a broader and more complex transcriptional reprogramming in tea plants, particularly involving stress-responsive lipid metabolism and secondary metabolite biosynthesis.

### 2.8. Differentially Expressed Transcription Factor Encoding Genes (TF-DEGs)

**Figure 7:**
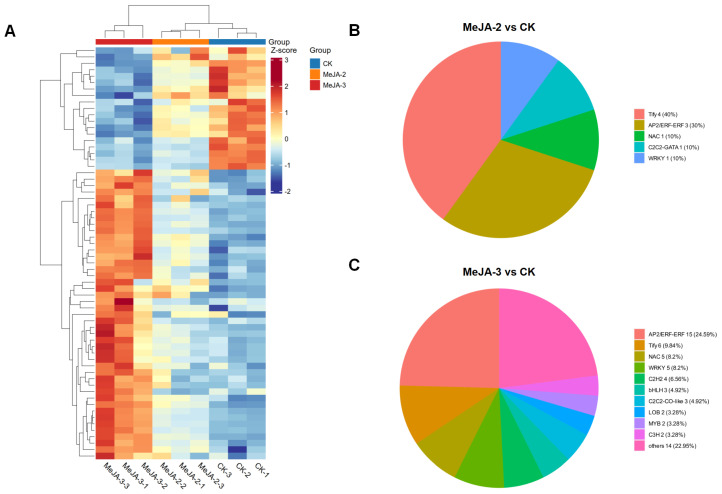
(**A**) Differentially expressed genes encoding transcription factors (TF-DEGs). (**B**) Classification of transcription factor families in the comparison between MeJA-2 and CK. (**C**) Classification of transcription factor families in the comparison between MeJA-3 and CK.

Transcription factors (TFs) play central regulatory roles in jasmonic acid (JA) signal transduction and the activation of plant defense responses. In this study, a total of 64 differentially expressed genes encoding transcription factors (TF-DEGs) were identified following MeJA treatment (Figure 7A). Under the MeJA-2 treatment, 10 TF-DEGs were significantly upregulated, including four members of the *TIFY/JAZ* family, three *AP2/ERF-ERF* family members, and one gene each from the *NAC*, *WRKY*, and *C2C2-GATA* families (Figure 7B). In contrast, the MeJA-3 treatment resulted in a much broader transcriptional response, with 61 TF-DEGs showing significant differential expression. These genes were predominantly distributed among the *AP2/ERF-ERF* (15 genes), *TIFY/JAZ* (6 genes), *NAC* (5 genes), *WRKY* (5 genes), *C2C2-GATA* (4 genes), *bHLH* (3 genes), and *MYB* (2 genes) families, along with several TFs from other families (Figure 7C).

Further comparative analysis revealed that four *TIFY/JAZ* family genes (*LJ118762*, *LJ044525*, *LJ111066*, and *LJ118019*), as well as one *NAC* gene (*LJ092126*) and one *WRKY* gene (*LJ049743*), were significantly upregulated under both MeJA-2 and MeJA-3 treatments. In contrast, three *AP2/ERF-ERF* genes (*LJ073691*, *LJ110457*, and *LJ110453*) and one *C2C2-GATA* gene (*LJ123281*) were consistently downregulated under both treatments.

Collectively, these results indicate that MeJA treatments of different intensities exert distinct regulatory effects on transcription factor expression in tea plants. The sustained induction of multiple *TIFY/JAZ* family transcriptional regulators across both MeJA concentrations suggests that they may represent core components of JA signaling, broadly participating in the initiation and modulation of defense responses. In contrast, the higher MeJA-3 treatment (2 mM) triggered differential expression of a wider range of TF families, particularly *AP2/ERF-ERF*, *NAC*, and *WRKY*, suggesting that these TFs may play more prominent roles in responding to high intensity defense signals and coordinating crosstalk among multiple signaling pathways.

### 2.9. Metabolic Enzymes DEGs

**Figure 8:**
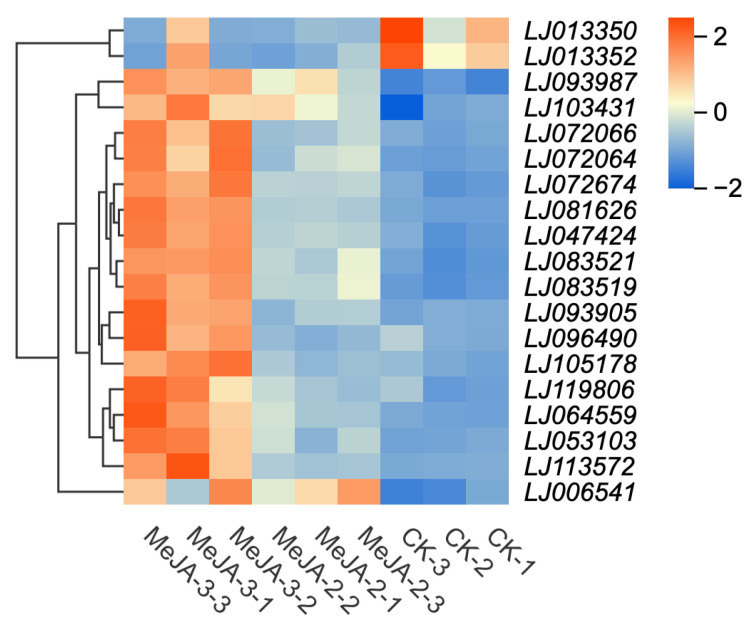
DEGs associated with metabolic enzymes.

In this study, a total of 20 DEGs associated with metabolic enzymes were identified (Figure 8), many of which were significantly upregulated in pathways related to plant defense and stress responses. Under both MeJA-2 and MeJA-3 treatments, seven DEGs were commonly induced. Among these shared genes, only the dammarenediol II synthase genes (*LJ013350* and *LJ013352*), involved in sesquiterpenoid and triterpenoid biosynthesis, were downregulated, whereas all other common DEGs exhibited an upward expression trend. The commonly upregulated genes included inositol oxygenase (*LJ072064* and *LJ072066*), which participates in ascorbate and aldarate metabolism, nucleotide sugar biosynthesis, and inositol phosphate metabolism; aldehyde dehydrogenase (*LJ006541*), involved in both ascorbate and aldarate metabolism and arginine and proline metabolism; and tryptophan synthase (*LJ081626* and *LJ064559*), which functions in phenylalanine, tyrosine, and tryptophan biosynthesis as well as glycine, serine, and threonine metabolism.

Under the MeJA-3 treatment, a larger set of genes associated with defense-related pathways was specifically induced. These genes primarily included eight DEGs involved in α-linolenic acid metabolism and linoleic acid metabolism, such as lipoxygenases (LOX) (*LJ083521*, *LJ083519*, and *LJ119806*) and allene oxide synthase (AOS) *CYP74A51* (*LJ093905*). In addition, genes encoding rhamnosyltransferases (*LJ105178* and *LJ096490*), associated with flavone and flavonol biosynthesis and flavonoid biosynthesis, were significantly upregulated. Furthermore, genes related to sesquiterpenoid and triterpenoid biosynthesis, including germacrene D synthase (*LJ113572*, *LJ053103*, and *LJ090544*), also showed marked induction under MeJA-3 treatment.

## 3. Discussion

Tea plants are continuously threatened by a wide range of herbivorous insects during their growth cycle, which represents a major constraint on the sustainable development and quality stability of the tea industry [39,40]. In this study, we evaluated the responses of tea plants to exogenous methyl jasmonate (MeJA) applied at different concentrations under field conditions, focusing on growth-related traits, quality-associated biochemical parameters, pest resistance performance, and transcriptome-level responses, thereby providing integrative phenotypic, biochemical, and molecular evidence for MeJA-mediated regulation in tea plants.

MeJA plays a critical role in plant defense signal transduction and the regulation of secondary metabolism. Previous studies have demonstrated that exogenous MeJA application can promote the accumulation of flavonoids and phenolic compounds in plants [41,42,43]. Consistent with these reports, our results showed that flavonoid content was significantly increased under MeJA treatments at concentrations ≤ 2 mM, whereas total phenolic content was markedly elevated under the 20 mM MeJA treatment. These compounds are widely recognized as important chemical defenses against herbivorous insects. Activation of defense responses is often accompanied by trade-offs between growth and primary metabolism [44]. In the present study, high-concentration MeJA (20 mM) significantly inhibited bud elongation and reduced chlorophyll content, indicating that excessive hormonal stimulation may suppress tea plant growth. By contrast, moderate MeJA concentrations (0.2–2 mM) did not significantly alter key quality-related biochemical components, such as free amino acids and soluble sugars, while being accompanied by distinct changes in shoot morphology, including shortened internodes, increased stem thickness, and reduced leaf insertion angles. These morphological traits are commonly regarded as favorable for the processing of flat-type green teas. It should be noted, however, that tea quality is a multifaceted concept encompassing not only biochemical composition but also sensory attributes such as bitterness, astringency, and aroma. Accordingly, the conclusions of this study are limited to the measured biochemical indices related to nutritional and processing-associated quality. Comprehensive sensory evaluations will be required in future studies to fully assess the influence of MeJA on overall tea quality.

MeJA treatment significantly enhanced field resistance of tea plants to *Empoasca onukii* and *Acaphylla theae*, as evidenced by a marked reduction in the population densities of both pests. This finding is consistent with the central role of jasmonates in plant defense against herbivorous insects and aligns with previous studies demonstrating that exogenous MeJA or JA can induce insect resistance in various crop species [45,46,47]. At the physiological level, MeJA treatments at concentrations ≥ 0.2 mM significantly increased the activity of PAL, while treatments at concentrations ≥ 2 mM also enhanced PPO activity, suggesting enhanced phenylpropanoid metabolism and oxidative defense capacity. Interestingly, MeJA treatments at concentrations ≥ 0.2 mM led to a reduction in SOD activity. Similar observations have been reported in slash pine, in which SOD activity was temporarily downregulated following infection by *Lecanosticta acicola* [48]. This phenomenon may reflect an active modulation of the antioxidant system, allowing transient accumulation of reactive oxygen species (ROS) that function as signaling molecules to activate downstream defense-related genes [49].

Under the MeJA-2 treatment, the number of differentially expressed genes (DEGs) was relatively limited, which may be attributable to the field-based experimental conditions, as field trials often yield fewer detectable DEGs than controlled indoor experiments while offering greater ecological relevance. Despite the limited DEG number, a targeted transcriptional response was evident, with enrichment in pathways related to defense priming and metabolic adjustment, including ascorbate and aldarate metabolism, terpenoid biosynthesis, aromatic amino acid biosynthesis, plant hormone signal transduction, and secondary metabolite biosynthesis. At this concentration, the transcriptional response was relatively concentrated and was mainly characterized by the upregulation of members of the *TIFY/JAZ* family, which may function as key regulators maintaining JA signaling homeostasis during defense activation. In contrast, the MeJA-3 treatment was associated with a substantially larger number of differentially expressed genes, accompanied by the broad activation of multiple core defense-related transcription factor families, including *AP2/ERF*, *NAC*, and *WRKY*. These transcription factors have been widely reported as central regulatory nodes involved in the integration of jasmonic acid signaling with other hormone pathways, such as salicylic acid and ethylene, and are generally implicated in the coordinated regulation of downstream defense-related gene expression [50,51]. Notably, under the MeJA-3 treatment, genes encoding key rate-limiting enzymes in the jasmonic acid biosynthesis pathway, including LOX and AOS, were significantly upregulated. The induction of these genes is commonly considered indicative of an enhanced capacity for endogenous JA biosynthesis and is consistent with the activation of JA-related signaling processes reported in previous studies [52,53]. In addition, the upregulation of genes involved in flavonoid biosynthesis and rhamnosyltransferases suggests a transcriptional shift toward the accumulation of secondary metabolites that are frequently associated with chemical defense against herbivores.

In conclusion, this study demonstrates that exogenous application of MeJA at appropriate concentrations (0.2–2 mM) can enhance field resistance of tea plants to major pests while simultaneously optimizing bud morphology for flat-type green tea processing. By integrating field phenotypes, biochemical traits, and transcriptomic data, this work advances the understanding of JA-mediated synergistic regulation of defense, growth, and metabolism in tea plants. Nevertheless, the long-term effects of MeJA application, cultivar-specific responses, and optimal application strategies under diverse cultivation conditions warrant further systematic investigation to facilitate its practical application in green tea plantation management.

## 4. Materials and Methods

### 4.1. Plant Materials, Sample Collection and Field Investigation

The cultivar *Camellia sinensis* ‘Zhongcha 108’ was used in this study. All field experiments were conducted during the spring growing season of 2025 at the Tea Germplasm Resource Nursery of Songyang County, Lishui City, Zhejiang Province, China, under uniform field conditions.

Following previous studies [54,55], five experimental groups were established: CK, MeJA-1 (0.02 mM), MeJA-2 (0.2 mM), MeJA-3 (2 mM), and MeJA-4 (20 mM), with 3 biological replicates in each group. Methyl jasmonate (MeJA) was first dissolved in dimethyl sulfoxide (DMSO) and then diluted with distilled water to the desired concentrations. Tween-20 was added as a surfactant at a final concentration of 0.01% (*v*/*v*), and the solutions were uniformly applied to tea leaves by foliar spraying. The control treatment (CK) received the same amount of DMSO and Tween-20 without MeJA. Each replicate maintained identical plot size and planting density, and consistent fertilizer and irrigation management were applied across all plots. Seven days after the initial MeJA application, for each replicate, several second leaves were randomly sampled, pooled, and divided into three portions: (i) for transcriptomic analysis and enzyme activity assays, the main vein was removed, and the samples were snap-frozen in liquid nitrogen and stored at −80 °C; (ii) for secondary metabolite analysis, leaves were de-veined, freeze-dried, ground to 60 mesh, and stored at −20 °C in the dark; (iii) for nutrient composition analysis, leaves were deactivated at 105 °C for 30 min, dried at 80 °C to constant weight, ground to 40 mesh, and stored in a desiccator at room temperature.

The second MeJA application was administered after a 7-day interval. Growth traits of one bud with two leaves, including bud length, internode length, and stem diameter, were measured 7 days after the second application. At the same time, pest population assessments targeting *Acaphylla theae* and *Empoasca onukii* were conducted. For each replicate, four tea plants were randomly selected, and the second leaf below the bud was collected from each plant, resulting in 12 leaves per treatment. The number of insects on each leaf was recorded.

### 4.2. Biochemical Parameter Assessment

Eight biochemical traits were selected for evaluation, including soluble sugars (SS), alkaloids, free amino acids (AA), flavonoids, phenylalanine ammonia-lyase (PAL), polyphenol oxidase (PPO), superoxide dismutase (SOD), and total phenols (TP). Amino acid content was determined using the ninhydrin colorimetric method. Soluble sugar content was measured using the anthrone colorimetric method. Flavonoid content was quantified using the Al(NO_3_)_3_ colorimetric assay. Alkaloid content was measured using the acid dye colorimetric method. Total phenol content was determined using the Folin–Ciocalteu method. The activities of superoxide dismutase (SOD; BC0175), phenylalanine ammonia-lyase (PAL; BC0215), and polyphenol oxidase (PPO; BC0195) were measured according to the instructions provided with the assay kits from Solarbio Life Sciences & Technology Co., Ltd. (Beijing, China).

### 4.3. RNA Extraction, Library Construction, Sequencing, and Read Alignment

Fresh second leaves (one bud with two leaves) were collected from the control, MeJA-2, and MeJA-3 treatments, with three biological replicates for each treatment. Total RNA was extracted using the Plant RNA Extraction Kit (DP441, Tiangen Biotech, Beijing, China), and genomic DNA contamination was removed using RNase-free DNase I. RNA quality was rigorously assessed using a NanoDrop 2000 spectrophotometer (Thermo Fisher Scientific, Wilmitong, DE, USA) for purity, a Qubit 4.0 fluorometer (Thermo Fisher Scientific, Waltham, MA, USA) for accurate quantification, and an Agilent 2100 Bioanalyzer (Agilent Technologies, Santa Clara, CA, USA) for RNA integrity. All RNA samples used for library preparation met the following criteria: OD260/280 of 1.8–2.2, OD260/230 ≥ 2.0, RNA integrity number (RIN) ≥ 7.0, and total RNA amount > 1 μg.

RNA-seq libraries were constructed using the VAHTS Universal V10 RNA-seq Library Prep Kit for Illumina (NR616-02, Vazyme Biotech Co., Ltd., Nanjing, China), following the manufacturer’s protocol. Briefly, poly(A) + mRNA was enriched using Oligo(dT) magnetic beads and fragmented at high temperature in fragmentation buffer. First-strand cDNA was synthesized using fragmented mRNA as the template, followed by second-strand cDNA synthesis. The resulting double-stranded cDNA was subjected to end repair, A-tailing, and adaptor ligation, and then amplified by PCR to generate the final libraries. Library quality and concentration were evaluated prior to sequencing.

The cDNA libraries were sequenced on an Illumina NovaSeq X platform (Illumina, Santa Clara, CA, USA) to generate paired-end reads. Raw sequencing data were processed using fastp (0.23.2) to remove low-quality reads and adaptor sequences. Clean reads were aligned to the *Camellia sinensis* reference genome (Assembly ID: GWHBQCR00000000, https://ngdc.cncb.ac.cn/gwh/Assembly/30226/show (accessed on 16 April 2025)) using HISAT2 (2.2.1) with default parameters. The reference genome was obtained from the NGDC Genome Warehouse.

### 4.4. Differentially Expressed Genes (DEGs) and Functional Enrichment Analysis

Transcript assembly was performed using StringTie (2.1.6), and the assembled transcripts were compared with the reference genome annotation using GffCompare (as implemented in the StringTie package) to identify novel transcripts. Gene-level raw read counts were generated using featureCounts. Differential expression analysis was conducted using DESeq2 (1.22.1), and genes with |log2(fold change)| > 1 and a false discovery rate (FDR) < 0.05 were defined as significantly differentially expressed genes (DEGs).

To investigate the biological functions of the DEGs, functional annotation was performed using DIAMOND against the Kyoto Encyclopedia of Genes and Genomes (KEGG) and Gene Ontology (GO) databases. KEGG pathway enrichment and GO term enrichment analyses were conducted, and terms with FDR < 0.05 were considered significantly enriched.

### 4.5. Data Analysis

All experimental data were statistically processed using Microsoft Excel 2019, and one-way analysis of variance (one-way ANOVA) was performed in SPSS 22.0 (IBM Corporation, 2013, Chicago, IL, USA) for datasets that met the assumptions of normality. Tukey’s HSD test was used for multiple comparisons (*p* < 0.05). Figures were generated using GraphPad Prism 9.0.

## 5. Conclusions

This study identifies a moderate MeJA concentration window (0.2–2 mM) under field conditions in which tea plants exhibit a favorable balance between defense activation and quality preservation. Within this range, MeJA application was associated with enhanced resistance to major pests without significantly altering key quality-related biochemical components, reflecting a “win–win” regulatory outcome. In addition to defense-related responses, MeJA treatments within this concentration window were accompanied by coordinated changes in shoot morphology that are commonly considered advantageous for mechanical handling and flat-type green tea processing, without evidence of quality trade-offs. At the mechanistic level, integrated transcriptomic and biochemical analyses indicate that this balanced response is linked to the synergistic regulation of jasmonate signaling, secondary metabolism, and stress-responsive pathways, highlighting a coordinated JA-mediated regulatory network in tea plants. Collectively, this work advances the understanding of how jasmonate signaling integrates defense, metabolism, and growth under field conditions, and provides a concrete concentration reference and practical basis for the application of plant immunity inducers in sustainable green tea plantation management.

## Figures and Tables

**Table 1:** Summary of Illumina RNA-sequencing data.

Samples	Clean Reads (Reads)	Clean Base (G)	GC Content (%)	Q30 (%)	Mapping Reads (%)
CK-1	66,075,638	10.06	45.41	96.79	91.24
CK-2	65,082,118	9.97	45.55	96.73	90.84
CK-3	82,571,356	12.60	43.65	97.03	91.81
MeJA-2-1	63,990,324	9.77	45.23	96.79	91.23
MeJA-2-2	67,252,488	10.26	45.28	96.72	91.37
MeJA-2-3	66,533,480	10.17	45.16	96.78	91.44
MeJA-3-1	63,788,598	9.72	45	96.78	91.55
MeJA-3-2	67,638,930	10.34	45.34	96.8	91.48
MeJA-3-3	76,421,390	11.62	44.78	96.79	91.49

## Data Availability

The transcriptome sequencing data was deposited in NCBI database under accession number: PRJNA1393621. https://www.ncbi.nlm.nih.gov/bioproject/PRJNA1393621 (accessed on 16 April 2025).

## Supplementary Materials

The following supporting information can be downloaded at: https://www.mdpi.com/article/10.3390/plants15020311/s1, Table S1: Differentially Expressed Genes in MeJA-2 vs. CK; Table S2: Differentially Expressed Genes in MeJA-3 vs. CK.

## References

[1] Mei Y. (2025). Analysis of China’s tea production and domestic sales in 2024. China Tea.

[2] Xiang F., Su Y., Zhou L., Dai C., Jin X., Liu H., Luo W., Yang W., Li W. (2025). Gibberellin promotes theanine synthesis by relieving the inhibition of *CsWRKY71* on *CsTSI* in tea plant (*Camellia sinensis*). Hortic. Res..

[3] Ma L.L., Lu S.F., Jin X.F., Gong Y. (2019). Effects of four kinds of plant growth regulators on the shoot growth and quality of *Camellia sinensis*. J. Tea Commun..

[4] Zhao X.P., Liu Y., Wei J.L., Chen X.M., Jiang T., Shi H., Xiao W.M., Ren Z.H., Wu H.H., Zhang H. (2024). Effects of plant growth regulators on growth and quality of spring tea shoots. J. Agric..

[5] Liechti R., Farmer E.E. (2002). The jasmonate pathway. Science.

[6] Wasternack C., Hause B. (2013). Jasmonates: Biosynthesis, perception, signal transduction and action in plant stress response, growth and development. Ann. Bot..

[7] Kim J., Chang C.R., Tucker M.L. (2015). To grow old: Regulatory role of ethylene and jasmonic acid in senescence. Front. Plant Sci..

[8] Wang H., Li Y., Pan J., Lou D., Hu Y., Yu D. (2017). The bHLH Transcription Factors *MYC2*, *MYC3*, and *MYC4* Are Required for Jasmonate-Mediated Inhibition of Flowering in *Arabidopsis*. Mol. Plant.

[9] Zhang F., Yao J., Ke J., Zhang L., Lam V.Q., Xin X.-F., Zhou X.E., Chen J., Brunzelle J., Griffin P.R. (2015). Structural basis of JAZ repression of MYC transcription factors in jasmonate signalling. Nature.

[10] Xiao S., Dai L., Liu F., Wang Z., Peng W., Xie D. (2004). COS1: An *Arabidopsis* coronatine insensitive1 Suppressor Essential for Regulation of Jasmonate-Mediated Plant Defense and Senescence. Plant Cell.

[11] Zhang L., Zou C., Zhu T., Du M., Zou X., He Y., Chen S., Long Q. (2024). The role of jasmonic acid in stress resistance of plants: A review. Chin. J. Biotechnol..

[12] Gui L.Y., Chen Z.M., Liu S.S. (2005). Effect of exogenous MJA treatment of tea plants on the growth of geometrid larvae. Sci. Agric. Sin..

[13] Pieterse C.M., Van der Does D., Zamioudis C., Leon-Reyes A., Van Wees S.C. (2012). Hormonal modulation of plant immunity. Annu. Rev. Cell Dev. Biol..

[14] Harb A., Krishnan A., Ambavaram M.M., Pereira A. (2010). Molecular and Physiological Analysis of Drought Stress in *Arabidopsis* Reveals Early Responses Leading to Acclimation in Plant Growth. Plant Physiol..

[15] Kang D.J., Seo Y.J., Lee J.D., Ishii R., Kim K.U., Shin D.H., Park S.K., Jang S.W., Lee I.J. (2005). Jasmonic acid differentially affects growth, ion uptake and abscisic acid concentration in salt-tolerant and salt-sensitive rice cultivars. J. Agron. Crop Sci..

[16] Wang Y., Tong W., Li F., Samarina L., Li P., Yang T., Zhang Z., Yi L., Zhai F., Wang X. (2024). LUX ARRHYTHMO links CBF pathway and jasmonic acid metabolism to regulate cold tolerance of tea plants. Plant Physiol..

[17] Hu Y., Jiang L., Wang F., Yu D. (2013). Jasmonate regulates the inducer of CBF expression–c-repeat binding factor/DRE binding factor1 cascade and freezing tolerance in *Arabidopsis*. Plant Cell.

[18] Chauvin A., Caldelari D., Wolfender J.L., Farmer E.E. (2013). Four 13-lipoxygenases contribute to rapid jasmonate synthesis in wounded *Arabidopsis thaliana* leaves: A role for lipoxygenase 6 in responses to long-distance wound signals. Annu. Rev. Plant Biol..

[19] Choi W.G., Hilleary R., Swanson S.J., Kim S.H., Gilroy S. (2016). Rapid, long-distance electrical and calcium signaling in plants. Annu. Rev. Plant Biol..

[20] Hilleary R., Gilroy S. (2018). Systemic signaling in response to wounding and pathogens. Curr. Opin. Plant Biol..

[21] Ali M.S., Baek K.-H. (2020). Jasmonic acid signaling pathway in response to abiotic stresses in plants. Int. J. Mol. Sci..

[22] Xu L., Liu F., Lechner E., Genschik P., Crosby W.L., Ma H., Peng W., Huang D., Xie D. (2002). The SCF^COI1^ ubiquitin-ligase complexes are required for jasmonate response in *Arabidopsis*. Plant Cell.

[23] Sheard L.B., Tan X., Mao H., Withers J., Ben-Nissan G., Hinds T.R., Kobayashi Y., Hsu F.-F., Sharon M., Browse J. (2010). Jasmonate perception by inositol-phosphate-potentiated COI1–JAZ co-receptor. Nature.

[24] Chini A., Fonseca S., Fernández G., Adie B., Chico J.M., Lorenzo O., García-Casado G., López-Vidriero I., Lozano F.M., Ponce M.R. (2007). The JAZ family of repressors is the missing link in jasmonate signalling. Nature.

[25] Wasternack C., Song S.S. (2017). Jasmonates: Biosynthesis, metabolism, and signaling by proteins activating and repressing transcription. J. Exp. Bot..

[26] Wasternack C., Strnad M. (2015). Jasmonate signaling in plant stress responses and development-active and inactive compounds. New Biotechnol..

[27] Tao J., Song X.F., Zhou Y.Y. (2021). Effects of MeJA on photosynthesis and secondary metabolism of *Forsythia suspensa* leaves. J. Henan Agric. Sci..

[28] Zhao X.P., Liu Y., Wei J.L., Chen X.M., Jiang T., Shi H. (2019). Effect of different concentration of methyl jasmonate on leaf photosynthesis and fluorescence of *Polygonum capitatum* Buch. -Ham. ex D. Don. J. Northeast Agric. Sci..

[29] Ahmadi F.I., Karimi K., Struik P.C. (2018). Effect of exogenous application of methyl jasmonate on physiological and biochemical characteristics of *Brassica napus* L. cv. Talaye under salinity stress. S. Afr. J. Bot..

[30] Yan Z., Wu Y.Q., Sun Y., Tang D.Q. (2018). Effects of exogenous methyl jasmonate on the growth of Freesia hybrida. J. Shanghai Jiaotong Univ. (Agric. Sci.).

[31] Yang T.Y., Han X.N., Xu Y.X., Tang J.X., Zhan K., Wu J.S., Wang L. (2020). Effects of foliar sprayed methyl jasmonate on potato minituber growth and yield. Subtrop. Plant Sci..

[32] Yang Y.C., Du Z.M., Zhang X.P., Li K.Y., Shen J.Q., Xu H. (2021). Effects of Spraying Methyl Jasmonate on Yield and Grain Quality of Japonica Rice during Heading and Flowering Stage. Crops.

[33] Salimi F., Shekari F., Hamzei J. (2016). Methyl jasmonate improves salinity resistance in German chamomile (*Matricaria chamomilla* L.) by increasing activity of antioxidant enzymes. Acta Physiol. Plant..

[34] Jiao L., Cai X.M., Bian L., Luo Z.X., Li Z.Q., Xi Z.J., Chen Z.M. (2018). Jasmonates: From induced plant anti-herbivore defensive reaction to growth-defense tradeoffs. Chin. J. Appl. Ecol..

[35] Cai X.M., Sun X.L., Dong W.X., Wang G.C., Chen Z.M. (2014). Herbivore species, infestation time, and herbivore density affect induced volatiles in tea plants. Chemoecology.

[36] Sun X.L., Dong W.X., Cai X.M., Gui L.Y., Chen Z.M. (2016). Variation in tea-plant volatiles induced by exogenous application of different concentrations of methyl jasmonate. Chin. J. Appl. Entomol..

[37] Qi T., Song S., Ren Q., Wu D., Huang H., Chen Y., Fan M., Peng W., Ren C., Xie D. (2011). The Jasmonate-ZIM-domain proteins interact with the WD-Repeat/bHLH/MYB complexes to regulate Jasmonate-mediated anthocyanin accumulation and trichome initiation in *Arabidopsis thaliana*. Plant Cell.

[38] Zhao M.L., Wang J.N., Shan W., Fan J.G., Kuang J.F., Wu K.Q., Li X.P., Chen W.X., He F.Y., Chen J.Y. (2013). Induction of jasmonate signalling regulators *MaMYC2s* and their physical interactions with *MaICE1* in methyl jasmonate-induced chilling tolerance in banana fruit. Plant Cell Environ..

[39] Hazarika L.K., Bhuyan M., Hazarika B.N. (2009). Insect pests of tea and their management. Annu. Rev. Entomol..

[40] Ye G.Y., Xiao Q., Chen M., Chen X.X., Yuan Z.J., Stanley D.W., Hu C. (2014). Tea: Biological control of insect and mite pests in China. Biol. Control.

[41] Guan Y.G., Hu W.Z., Jiang A.L., Xu Y.P., Sa R.G.W., Feng K., Zhao M.R., Yu J.X., Ji Y.R., Hou M.Y. (2019). Effect of methyl jasmonate on phenolic accumulation in wounded broccoli. Molecules.

[42] Zhang X.X., Hong B., Jing L.L., Cao S., Jia Y.X. (2020). Insect resistance of tomato induced by exogenous methyl jasmonate to *Bemisia tabaci*. Chin. J. Ecol..

[43] Ali M.B., Hahn E.J., Paek K.Y. (2007). Methyl jasmonate and salicylic acid induced oxidative stress and accumulation of phenolics in *Panax ginseng* bioreactor root suspension cultures. Molecules.

[44] Züst T., Agrawal A.A. (2017). Trade-offs between plant growth and defense against insect herbivory: An emerging mechanistic synthesis. Annu. Rev. Plant Biol..

[45] Wang J.B., Wang H.F., Cui X.Y., Yang Y.T. (2010). Inducement of methyl jasmonate on resistance of tobacco to cotton bollworms. Chin. Agric. Sci. Bull..

[46] Wu X.X., He J., Zhou F.C., Chen X.H., Yang A.M., Zhang H.B. (2019). Physiological responses of different whitefly resistant peppers to exogenous methyl jasmonate. Chin. J. Ecol..

[47] Wang Y., Liu C.J., Zhang X., Li W.L., Wang F. (2021). Inhibitory effect of methyl jasmonate on fusarium crown and root rot of tomato. North. Hortic..

[48] Cheng F., Ye J.R., Liu G., An H.C. (2012). Study on PAL, PPO, SOD activities of tissue cultured plantlets of slash pine treated with *Lecanosticta acicola*. Forest Res..

[49] Mittler R. (2017). ROS are good. Trends Plant Sci..

[50] Li N., Han X., Feng D., Yuan D., Huang L.J. (2019). Signaling crosstalk between salicylic acid and ethylene/jasmonate in plant defense: Do we understand what they are whispering?. Int. J. Mol. Sci..

[51] Zhou J.G., Mu Q., Wang X.Y., Zhang J., Yu H.Z., Huang T.Z., He Y.X., Dai S.J., Meng X.Z. (2022). Multilayered synergistic regulation of phytoalexin biosynthesis by ethylene, jasmonate, and MAPK signaling pathways in *Arabidopsis*. Plant Cell.

[52] Laudert D., Weiler E.W. (1998). Allene oxide synthase: A major control point in *Arabidopsis thaliana* octadecanoid signalling. Plant J..

[53] Ghorbel M., Brini F., Sharma A., Landi M. (2021). Role of jasmonic acid in plants: The molecular point of view. Plant Cell Rep..

[54] Das S., Goswami M., Yadav R.N.S., Baruah A.M., Bandyopadhyay T. (2024). Methyl jasmonate alters expression of enzymes and metabolites of terpenoid biosynthesis in tea cell culture. Plant Cell Tissue Organ Cult..

[55] Shi J., Ma C.Y., Qi D.D., Lv H.P., Yang T., Peng Q.H., Chen Z.M., Lin Z. (2015). Transcriptional responses and flavor volatiles biosynthesis in methyl jasmonate-treated tea leaves. BMC Plant Biol..

